# Regulatory T cell dysfunction and exhaustion in uveitis: immunometabolic mechanisms, microenvironmental drivers, and emerging therapeutic strategies

**DOI:** 10.3389/fimmu.2026.1793865

**Published:** 2026-05-05

**Authors:** Xingyu Su, Qiuyu Tan, Liu Zheng, Zhixiang Ding

**Affiliations:** Department of Ophthalmology, The First Affiliated Hospital of Guilin Medical University, Guilin, China

**Keywords:** autoimmune, molecular mechanisms, regulatory T cells (Tregs), Th17/Treg balance, therapeutic strategies, TIGIT, uveitis

## Abstract

Non-infectious uveitis encompasses a diverse array of autoimmune ocular disorders marked by the breakdown of immune tolerance and recurrent inflammatory episodes. Regulatory T cells (Tregs) are integral to the maintenance of ocular homeostasis in all subtypes of this condition. However, Behçet’s disease, Vogt-Koyanagi-Harada (VKH) disease, and HLA-B27-associated uveitis exhibit distinct patterns of Treg quantitative reduction, lineage instability, and functional exhaustion. Tregs play a crucial role in maintaining ocular homeostasis; however, their quantitative reduction, lineage instability, and functional exhaustion significantly contribute to the persistence of the disease. This review provides a systematic synthesis of the molecular and immunometabolic mechanisms underlying Treg exhaustion in uveitis, emphasizing both universal pathways and subtype-specific mechanisms. We examine critical intrinsic regulators of Treg fitness, including the multidimensional control of FoxP3 stability, the upregulation of inhibitory checkpoints such as TIGIT and PD-1, and the dysregulated plasticity within the Th17/Treg axis. Additionally, the review emphasizes how extrinsic microenvironmental factors influence Treg functionality, with a specific focus on adenosine A2A receptor (A2Ar) signaling, circadian rhythm disruption mediated by the clock gene Per1, and the immunomodulatory role of gut microbiota. Regarding therapeutic strategies, we evaluate recent advances in restoring Treg competence, including immune checkpoint agonists, metabolic reprogramming agents (e.g., Itaconate), and traditional herbal formulations.

## 1 Introduction

Uveitis, characterized by intraocular inflammation of the uveal tract, is a major contributor to visual impairment and blindness globally. This condition comprises a diverse group of diseases with varied etiologies, including infectious agents and non-infectious autoimmune processes. Non-infectious uveitis is often associated with systemic autoimmune diseases or may occur as an isolated ocular disorder. Notably, uveitis is responsible for approximately 10-15% of cases of blindness in developed countries, predominantly affecting individuals of working age, thus imposing significant socio-economic burdens due to reduced productivity and diminished quality of life (1). The clinical manifestations of uveitis are highly variable, ranging from mild symptoms to severe inflammation, which can lead to complications such as cataracts, glaucoma, macular edema, and ultimately irreversible vision loss (1). Although there have been advances in immunosuppressive therapies, the management of uveitis remains challenging due to its chronic, relapsing nature and the potential for treatment-related adverse effects. Non-infectious uveitis encompasses a variety of forms categorized by anatomical location (anterior, intermediate, posterior, panuveitis), clinical progression (acute, recurrent, chronic), and systemic associations such as Behçet’s disease and sarcoidosis. Immune profiles may vary between peripheral blood and intraocular fluids or tissues. Consequently, it is essential to specify the source of evidence, whether from peripheral blood mononuclear cells (PBMC), aqueous humor/vitreous, or ocular infiltrates, and to interpret “Treg exhaustion/dysfunction” with consideration of the specific phase of the disease.

Non-infectious uveitis comprises a diverse array of conditions characterized by distinct immunopathological mechanisms. Prominent subtypes include Behçet’s disease (BD), Vogt-Koyanagi-Harada (VKH) disease, and HLA-B27-associated uveitis, each distinguished by unique T cell phenotypes, cytokine profiles, and metabolic signatures. Kang et al.’s single-cell analysis demonstrated differential T cell infiltration and clonal expansion between VKH and BD (2). Additionally, HLA-B27-associated uveitis is marked by a distinct Th17/Treg imbalance (3). These findings indicate that mechanisms of Treg exhaustion may vary by subtype, with significant implications for personalized therapeutic approaches. Nonetheless, it is important to note that many mechanistic studies rely on experimental autoimmune uveitis (EAU) models, which may not adequately reflect the heterogeneity of human disease, a limitation acknowledged throughout this review.

The pathogenesis of autoimmune uveitis is fundamentally associated with the disruption of immune homeostasis, particularly involving T lymphocyte subsets. Within this context, regulatory T cells (Tregs), characterized by the expression of the transcription factor Foxp3, are crucial for maintaining immune tolerance by suppressing inappropriate immune responses and preventing tissue damage (4). Tregs execute their immunosuppressive functions through various mechanisms, including the secretion of anti-inflammatory cytokines, modulation of antigen-presenting cells, and direct suppression of effector T cells (5). The equilibrium between pro-inflammatory T helper 17 (Th17) cells and Tregs is particularly vital; disturbances in this balance have been implicated in the onset and persistence of ocular inflammation in uveitis (4, 6). Importantly, Tregs not only facilitate the resolution of inflammation but also influence disease recurrence by modulating immune responses within the ocular microenvironment (4).

Recent studies have increasingly concentrated on elucidating the molecular mechanisms responsible for Treg dysfunction or depletion in uveitis, underscoring their significance in disease pathogenesis and potential as therapeutic targets. Treg exhaustion or functional impairment, marked by altered expression of surface molecules such as CTLA-4, PD-1, and TIGIT, has been identified as a contributing factor to persistent ocular inflammation (7–9). Notably, CTLA-4 expression on Tregs is crucial for the suppression of autoimmune uveitis, with distinct requirements observed between circulating and ocular-resident Tregs (7). Furthermore, metabolic dysregulation and signaling pathways, including PI3K/AKT, NF-κB, and TGF-β, have been implicated in the modulation of Treg stability and suppressive function in experimental uveitis models (6, 10). The dynamic interactions between Tregs and other immune cells, such as Th17 cells and mucosal-associated invariant T (MAIT) cells, further shape the inflammatory environment and influence clinical outcomes (11).

In therapeutic contexts, strategies focused on reestablishing regulatory T cell (Treg) homeostasis or enhancing their functionality have demonstrated potential in both preclinical and clinical environments. These strategies encompass low-dose interleukin-2 therapy to selectively expand Tregs, the adoptive transfer of ex vivo-expanded Tregs, and the modulation of immune checkpoints to counteract Treg exhaustion (12, 13). Innovative delivery systems, such as hyaluronan methylcellulose hydrogels, have been developed to enhance the survival and stability of transferred Tregs within inflamed ocular tissues (13). Furthermore, targeting specific molecular pathways implicated in Treg depletion, such as the Wnt/β-catenin axis and glutathione peroxidase 4-mediated ferroptosis, presents promising avenues for therapeutic intervention (14, 15). The identification of Treg subsets with distinct phenotypic and functional characteristics, including Immune Regulatory 1 cells (IR1) and TIRC7-expressing Tregs, offers additional insights for optimizing Treg-based therapies (16, 17).

In this review, we employ the term “Treg dysfunction” as a comprehensive category that includes quantitative depletion, impaired trafficking, unstable lineage identity, and diminished suppressive function. Conversely, we define “Treg exhaustion” more specifically as a state induced by chronic inflammation, characterized by persistent expression of inhibitory receptors (e.g., PD-1/TIGIT), metabolic insufficiency, epigenetic and lineage instability (such as FOXP3 destabilization), and a decline in suppressive capacity, particularly in the context of continuous antigenic and cytokine stimulation. It is crucial to note that the expression of markers alone does not equate to exhaustion; rather, exhaustion should be assessed in relation to the disease phase, tissue localization, and functional outcomes.

**Figure 1:**
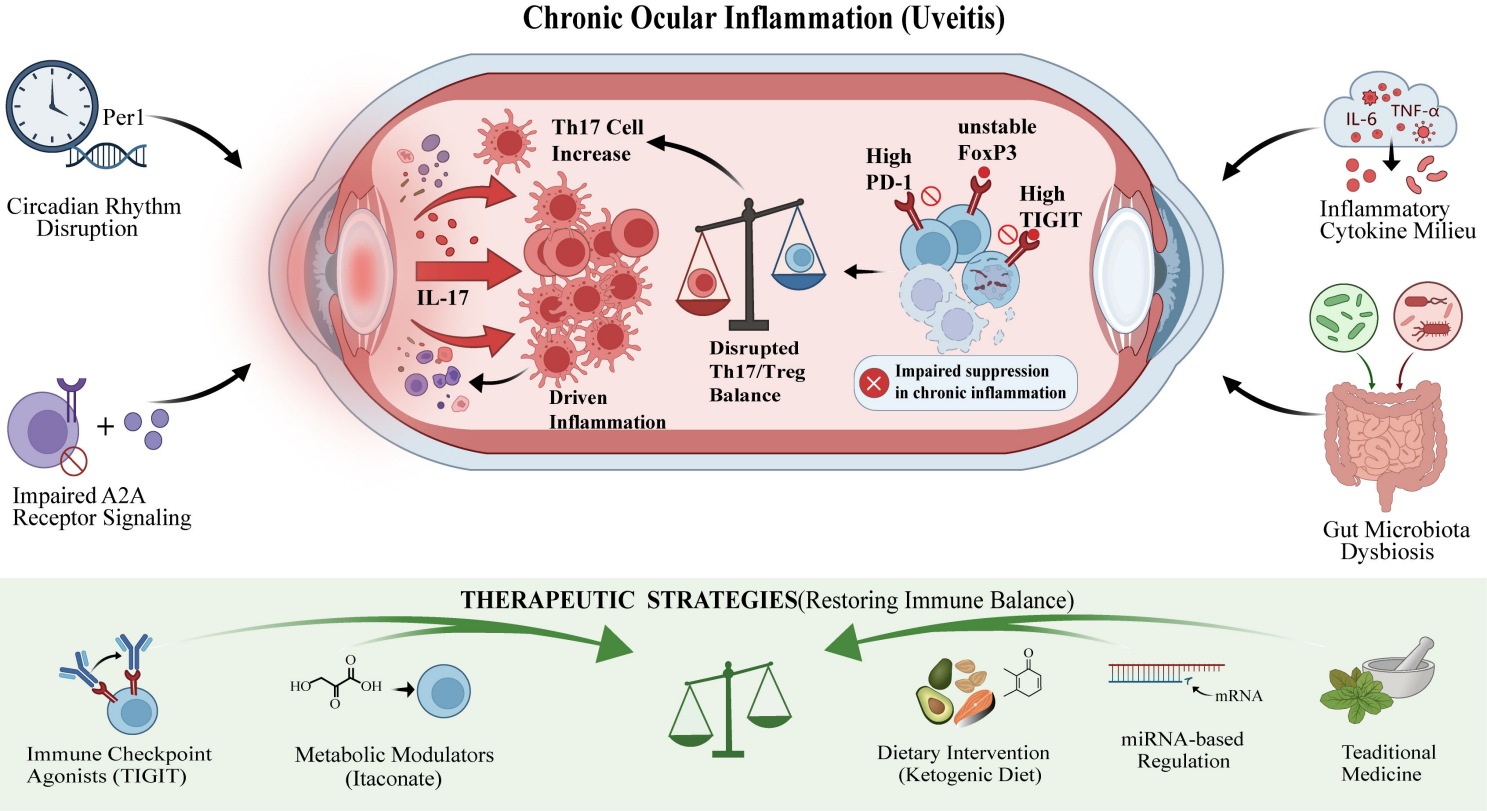
Integrated model of regulatory T cell (Treg) dysfunction/exhaustion in chronic uveitis and emerging Treg-centered therapeutic opportunities. The schematic summarizes convergent intrinsic and extrinsic mechanisms proposed to drive persistent ocular inflammation in non-infectious uveitis by destabilizing the Treg program and disrupting the Th17/Treg equilibrium. Within the inflamed eye, chronic antigen exposure and an inflammatory cytokine milieu (e.g., IL−6 and TNF−α) are associated with Th17 expansion and increased IL−17 production, while Tregs exhibit an exhaustion-like phenotype characterized by high PD−1 and TIGIT expression, FoxP3 instability, and impaired suppressive function, collectively resulting in a shift toward Th17 dominance and sustained tissue inflammation. Systemic/microenvironmental drivers highlighted in this review include circadian rhythm disruption (linked to reduced Per1 and metabolic insufficiency in Tregs), impaired adenosine A2Ar signaling (limiting induction/maintenance of suppressive Treg subsets, including A2Ar-dependent TIGIT+Tregs), and gut microbiota dysbiosis, which amplifies inflammatory mediators and supports pathogenic Th17 polarization that can traffic to the eye. The lower panel outlines representative therapeutic concepts aimed at restoring immune balance, including checkpoint agonism (e.g., TIGIT stimulation), immunometabolic modulators (e.g., itaconate), dietary/metabolic interventions (e.g., ketogenic diet), miRNA-based regulation, and traditional medicine-derived approaches, which are proposed to reinforce Treg fitness and/or restrain Th17-driven pathology. This figure is a conceptual summary of mechanisms and translational strategies discussed in the manuscript and does not imply that any single pathway is solely responsible for disease in all uveitis endotypes. A2Ar, adenosine A2A receptor; FoxP3, forkhead box P3; IL, interleukin; PD-1, programmed cell death protein 1; Per1, period circadian regulator 1; Th17, T helper 17 cell; TIGIT, T cell immunoreceptor with immunoglobulin and ITIM domains; TNF-α, tumor necrosis factor alpha; Treg, regulatory T cell.

In conclusion, uveitis is a multifaceted autoimmune disorder in which Tregs are essential for maintaining ocular immune privilege and preventing pathological inflammation. The depletion or dysfunction of Tregs is closely associated with the onset, progression, and recurrence of uveitis. Recent advances in elucidating the molecular mechanisms that regulate Treg homeostasis, exhaustion, and their interactions with other immune components have led to the development of innovative therapeutic strategies. This review seeks to systematically consolidate current knowledge regarding the molecular mechanisms underlying Treg depletion in uveitis and to explore emerging Treg-based therapeutic approaches, thereby promoting clinical translation and the development of novel treatments for this sight-threatening condition (**Figure 1**). In the sections that follow, we consistently enhance conceptual continuity by outlining each exhaustion-related mechanism, summarizing uveitis-specific evidence (from human samples and EAU), and discussing therapeutic implications and knowledge gaps.

## 2 Immunoregulatory role and depletion mechanism of Tregs in uveitis

### 2.1 FoxP3 stability and maintenance of Tregs function

FoxP3 serves as the essential transcription factor defining the lineage of Tregs, playing an essential role in their development, identity, and suppressive function. The persistent expression of FoxP3 is critical for sustaining the suppressive capacity of Tregs and maintaining immune homeostasis. A loss or instability in FoxP3 expression results in the formation of “exFoxP3” cells, which lose their suppressive capabilities and may differentiate into pro-inflammatory effector T cells, thereby contributing to the pathogenesis of autoimmune diseases. Operating at transcriptional, post-transcriptional, and post-translational levels, the molecular mechanisms that regulate FoxP3 stability collectively ensure the integrity of Treg lineage commitment and function.

At the transcriptional level, conserved non-coding sequences (CNS), such as CNS0 and CNS3 located upstream of the FoxP3 promoter, are integral in orchestrating the initiation and maintenance of FoxP3 expression. The deletion of these enhancer elements disrupts the generation of thymic Tregs and compromises FoxP3 stability in peripheral Tregs, ultimately leading to systemic autoimmunity. This highlights their essential role in Treg development and lineage stability (18). Epigenetic regulation is also crucial; the demethylation of the Treg-specific demethylated region (TSDR) within the FoxP3 locus is necessary for stable FoxP3 expression. Although targeted demethylation of the TSDR induces physiological FoxP3 expression, it is insufficient by itself to fully establish the Treg suppressive phenotype, suggesting that additional factors are required to maintain Treg identity (19). Moreover, the transcription factor BATF has been identified as vital for Treg homeostasis and stability. It regulates FoxP3 expression and facilitates the demethylation of CNS2, a critical enhancer region, thereby preventing autoimmune pathology (20).

Post-transcriptional regulation is mediated by RNA-binding proteins, such as HuR, which directly interact with and stabilize FoxP3 mRNA, thereby preventing its degradation. The deletion of HuR in Tregs leads to diminished stability of FoxP3 mRNA, disruption of T helper cell differentiation pathways, and compromised suppressive function. This underscores the critical role of mRNA stability in sustaining FoxP3 expression and Treg functionality (21). Furthermore, heterogeneous nuclear ribonucleoprotein A1 (hnRNPA1) associates with both the FoxP3 protein and the E3 ligase Stub1, thereby enhancing FoxP3 stability by modulating its ubiquitination status. The absence of hnRNPA1 results in increased ubiquitination of FoxP3 and a subsequent decline in Treg suppressive capacity (22).

Post-translational modifications play a central role in modulating the stability and functionality of the FoxP3 protein. The processes of ubiquitination and deubiquitination serve as dynamic regulators of FoxP3 protein turnover. The E3 ubiquitin ligase MDM2 is responsible for both monoubiquitination and polyubiquitination of FoxP3, which paradoxically leads to the stabilization of the protein and an enhancement of regulatory T cell (Treg) suppressive function. The knockdown of MDM2 results in the destabilization of FoxP3 and a subsequent impairment of Treg function, whereas its overexpression contributes to increased FoxP3 stability (23). In a similar vein, the deubiquitinase USP44, in conjunction with USP7, facilitates the removal of K48-linked ubiquitin chains from FoxP3, thereby preventing its degradation via the proteasome and maintaining Treg function during inflammatory conditions (24). In contrast, signaling through the insulin receptor substrate 1 (IRS1) activates mTORC1, which exerts a negative regulatory effect on FoxP3 expression and Treg stability, ultimately leading to Treg destabilization and a reduction in suppressive function (25).

Metabolic regulation is intricately linked to the stability of FoxP3. Acetyl-CoA carboxylase 1 (ACC1) functions as a dual metabolic-epigenetic regulator by influencing lipid synthesis and protein acetylation, which in turn facilitates FoxP3 transcription and the differentiation of Tregs. Inhibition of ACC1 leads to increased histone and protein acetylation, thereby maintaining FoxP3 expression and enhancing the suppressive capacity of Tregs even in inflammatory environments (26). These observations underscore the critical role of metabolic signals in the regulation of FoxP3 and the stability of Tregs.

The signaling pathway of the adenosine A2A receptor (A2Ar) has been demonstrated to facilitate the induction and stabilization of FoxP3+ Tregs. Disruption of this pathway is evident in patients with autoimmune uveitis, indicating a mechanistic association between impaired A2Ar signaling, FoxP3 instability, and Treg dysfunction in the context of autoimmune ocular inflammation (23). Furthermore, the DEL-1/αvβ3 integrin axis enhances RUNX1-dependent FoxP3 expression and promotes demethylation of the TSDR, thereby stabilizing FoxP3 expression and enhancing Treg function at barrier sites. This axis presents potential therapeutic opportunities for the treatment of inflammatory and autoimmune diseases (27).

FoxP3 is expressed in various isoforms produced through alternative splicing, each possessing distinct functions and regulatory mechanisms. The full-length isoform is essential for preserving the stability of the Treg lineage, whereas isoforms lacking exon 2 are linked to diminished suppressive function and the emergence of autoimmune phenotypes. The equilibrium among these isoforms is modulated by metabolic and signaling pathways, and their dysregulation is implicated in disease pathogenesis (28, 29). The enforced overexpression of FoxP3, including in chimeric antigen receptor (CAR)-Tregs, enhances both stability and suppressive function without leading to exhaustion or dysfunction, highlighting the therapeutic potential of manipulating FoxP3 levels to sustain Treg identity (30, 31).

**Figure 2:**
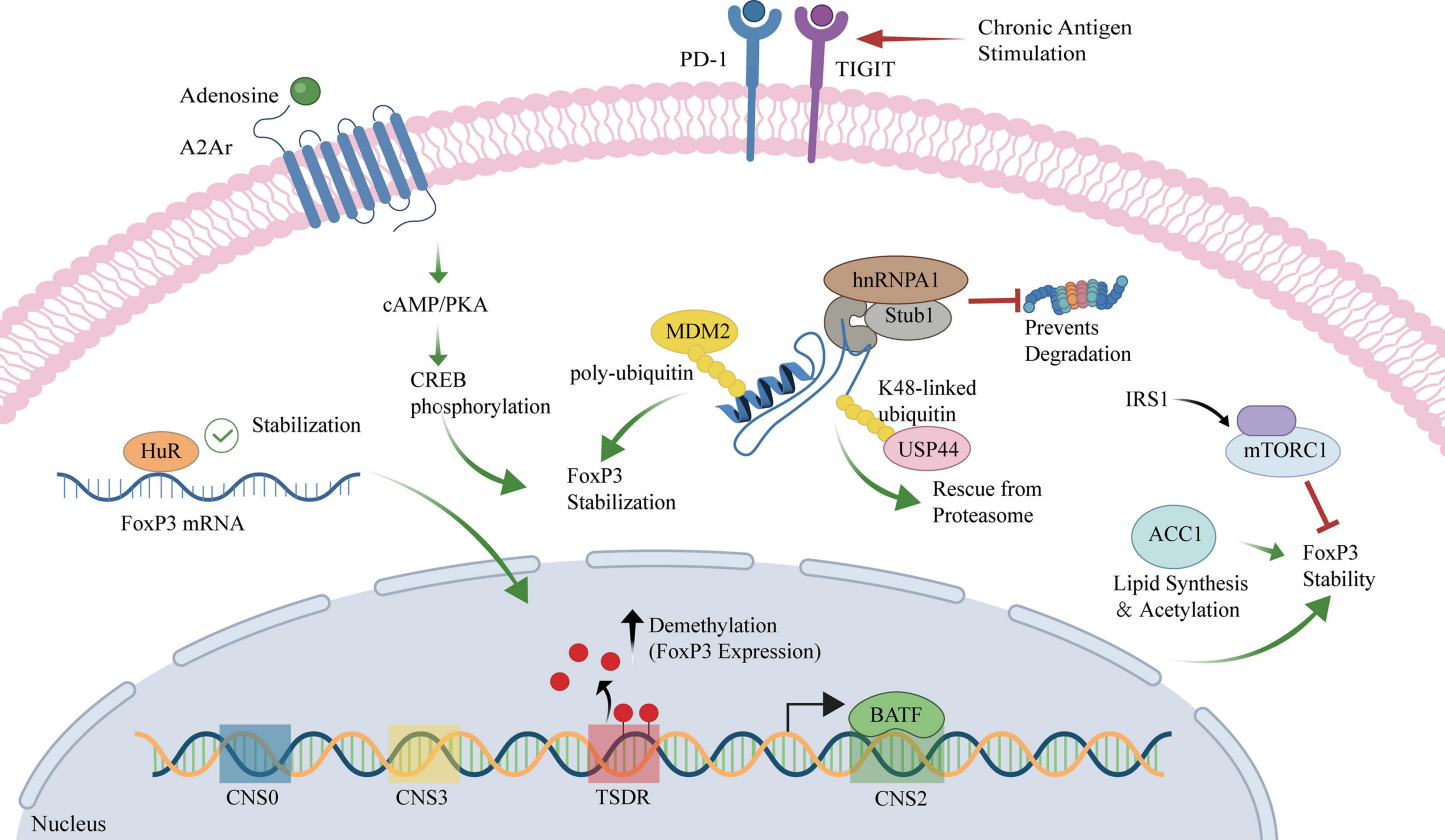
Intracellular networks driving Treg exhaustion and FoxP3 instability. Schematic overview of key cell-intrinsic pathways that preserve or erode FoxP3 stability in Tregs, integrating membrane receptor signaling, post-transcriptional and post-translational regulation, metabolic control, and nuclear epigenetic/transcriptional programs. At the plasma membrane, adenosine engagement of the adenosine A2Ar activates the cAMP/PKA axis, leading to CREB phosphorylation and nuclear translocation, thereby promoting FoxP3 stabilization. In parallel, chronic antigen stimulation is linked to sustained expression of inhibitory checkpoints (PD−1 and TIGIT) associated with an exhaustion-like state. In the cytoplasm, the RNA-binding protein HuR binds FoxP3 mRNA to enhance transcript stability. FoxP3 protein abundance is further tuned by the ubiquitination/deubiquitination machinery: the E3 ligase MDM2 mediates polyubiquitination in a manner associated with FoxP3 stabilization; hnRNPA1 cooperates with Stub1 to prevent FoxP3 degradation; and the deubiquitinase USP44 removes K48-linked ubiquitin chains to rescue FoxP3 from proteasomal turnover. Metabolically, the IRS1–mTORC1 pathway negatively regulates FoxP3 stability, whereas ACC1-driven lipid synthesis and acetylation-related processes support FoxP3 stabilization. In the nucleus, FoxP3 regulatory elements (CNS0/CNS3, CNS2, and the Treg-specific demethylated region, TSDR) are highlighted; TSDR demethylation correlates with increased FoxP3 expression and lineage stability, and BATF binding at CNS2 contributes to transcriptional control. Green arrows indicate activation/stabilization, and red blunt-ended lines indicate inhibition/degradation. A2Ar, adenosine A2A receptor; ACC1, acetyl-CoA carboxylase 1; BATF, basic leucine zipper ATF-like transcription factor; cAMP, cyclic adenosine monophosphate; CNS, conserved non-coding sequence; CREB, cAMP response element-binding protein; FoxP3, forkhead box P3; hnRNPA1, heterogeneous nuclear ribonucleoprotein A1; HuR, human antigen R; IRS1, insulin receptor substrate 1; MDM2, mouse double minute 2 homolog; mTORC1, mechanistic target of rapamycin complex 1; PD-1, programmed cell death protein 1; PKA, protein kinase A; Stub1, STIP1 homology and U-box containing protein 1; TIGIT, T cell immunoreceptor with immunoglobulin and ITIM domains; TSDR, Treg-specific demethylated region; USP44, ubiquitin-specific peptidase 44.

A schematic overview of convergent pathways governing FOXP3 stability and their potential links to Treg dysfunction/exhaustion is provided in **Figure 2**.

In conclusion, the stable expression of FoxP3 is a complex process governed by enhancer elements, epigenetic modifications, mRNA stability, post-translational modifications, metabolic signals, and isoform balance. Disruption at any of these regulatory nodes can result in FoxP3 instability, Treg dysfunction, and the emergence of pathogenic “exFoxP3” cells, thereby contributing to autoimmune inflammation, such as uveitis. Within the inflamed eye, persistent IL-6/IL-1β exposure and hypoxic/metabolic stress can destabilize FOXP3 and promote exFoxP3 emergence, weakening local immune privilege. Therapeutic strategies aimed at enhancing FoxP3 stability—by targeting ubiquitination pathways, metabolic checkpoints, or signaling pathways such as A2Ar—show potential for restoring Treg function and treating autoimmune diseases.

### 2.2 The role of immune checkpoint molecules TIGIT and PD-1 in Tregs exhaustion

TIGIT (T cell immunoreceptor with immunoglobulin and ITIM domains) and PD-1 (programmed cell death protein 1) are inhibitory receptors prominently expressed on Tregs, playing critical roles in modulating their immunosuppressive functions. Both molecules serve as immune checkpoints that contribute to the maintenance of immune homeostasis by restraining excessive immune activation, but their dysregulation is implicated in pathological immune exhaustion, including in autoimmune and inflammatory diseases such as uveitis. TIGIT is highly expressed on activated T cells, including Tregs, natural killer (NK) cells, and tumor-infiltrating lymphocytes, where it mediates inhibitory signaling through its interaction with ligands such as CD155, leading to suppression of effector T cell responses and enhancement of Treg-mediated immunosuppression (32, 33). PD-1 similarly functions as a negative regulator of T cell activation, and its expression on Tregs contributes to their suppressive capacity and exhaustion phenotype (34). TIGIT and PD-1 expression on Tregs shouldn’t be seen as a uniform “exhaustion signature”. Inflammatory conditions might cause temporary upregulation, indicating an active, suppressive Treg state, especially during resolution. A true exhaustion phenotype involves persistent co-expression of multiple inhibitory receptors, functional impairment, metabolic insufficiency, and FOXP3 instability in chronic or relapsing diseases (35, 36). Therefore, in uveitis/EAU, checkpoint receptor expression should be evaluated alongside functional assays, lineage stability, and metabolic/epigenetic health, not just by expression levels.

Research in oncology has identified TIGIT/PD-1-high Tregs as highly suppressive populations influenced by the tumor microenvironment (36). However, caution is warranted when extrapolating these findings to uveitis, as the autoimmune ocular environment is characterized by distinct antigen sources, inflammatory dynamics, and therapeutic objectives. In the context of uveitis, diminished Treg suppressive function and lineage instability contribute to pathogenesis, whereas in cancer, attenuating Treg suppression may enhance anti-tumor immunity. Consequently, this review prioritizes data specific to uveitis and EAU, while oncology findings are considered primarily as mechanistic hypotheses, acknowledging their inherent limitations.

The TIGIT+ Treg subset has been associated with remission phases in inflammatory diseases, including uveitis, highlighting its role in disease modulation. Activation of TIGIT signaling through agonists has been shown to suppress Th17 cell infiltration and attenuate inflammation, suggesting that TIGIT+ Tregs contribute to the resolution of immune-mediated tissue damage (37). This is supported by evidence from cancer immunology, where TIGIT expression delineates Tregs with enhanced suppressive function and exhaustion markers, and TIGIT blockade can modulate Treg activity and tumor immunity (32, 33).

PD-1+ and TIGIT+ Tregs migrate to sites of inflammation in a chemokine receptor-dependent manner, with CCR6 and CCR7 playing pivotal roles in their tissue homing. Aberrant expression of these chemokine receptors can disrupt Treg localization and function, thereby impairing immune regulation at inflammatory sites such as the uveal tract (38). The co-expression of TIGIT and PD-1 on Tregs is often observed in exhausted T cell populations within inflamed tissues, where these cells exhibit diminished effector functions but sustained immunosuppressive activity (39). This dual expression pattern is linked to a regulatory phenotype characterized by high levels of inhibitory receptors and cytokines such as IL-10 and TGF-β, which are essential for maintaining immune tolerance and preventing tissue damage.

Mechanistically, TIGIT engagement on Tregs can induce antibody-dependent cellular cytotoxicity (ADCC) and reduce dendritic-cell co-stimulation (CD80/CD86) and inflammatory cytokine production, thereby limiting Th17 priming and shaping the immune microenvironment (40). The functional interplay between TIGIT and PD-1 pathways suggests that combined targeting of these checkpoints may enhance therapeutic efficacy by modulating Treg exhaustion and restoring immune balance (41). Moreover, the expression of TIGIT and PD-1 on Tregs correlates with clinical outcomes and disease activity in various contexts, underscoring their potential as biomarkers and therapeutic targets (42, 43).

TIGIT and PD-1 are crucial inhibitory receptors on Tregs, influencing their suppressive roles based on context. The TIGIT+ Treg subset is linked to disease remission in uveitis, indicating a suppressive function, whereas chronic co-expression may lead to dysfunction. Activating TIGIT signaling can reduce inflammation by curbing Th17 cell infiltration, likely by strengthening TIGIT+ Tregs.

### 2.3 Disruption of Th17/Treg cell balance and its impact

The balance between Th17 cells and Tregs represents a dynamic equilibrium crucial for maintaining immune homeostasis in uveitis, particularly autoimmune uveitis (AU) and its experimental model, experimental autoimmune uveitis (EAU). Th17 cells drive inflammation through interleukin-17 (IL-17) production, which promotes tissue damage, whereas Tregs counteract this by suppressing immune responses and maintaining tolerance. In uveitis, this balance is often disrupted, resulting in an increased frequency and pathogenicity of Th17 cells alongside a reduction in Treg numbers and function, which collectively contribute to persistent ocular inflammation and tissue injury.

Multiple studies have consistently demonstrated that in both human uveitis patients and EAU models, there is a significant elevation of Th17 cells and their associated cytokines, such as IL-17A, coupled with a concomitant decrease in Treg populations and their suppressive capacity. For example, flow cytometric analyses of aqueous humor and peripheral blood mononuclear cells (PBMCs) from uveitis patients reveal a marked increase in Th17 cells and a decrease in Tregs, correlating with elevated IL-17 and reduced IL-10 expression, indicating an inflammatory milieu favoring disease progression (44). Similarly, in EAU models, the peak of intraocular inflammation coincides with increased Th17 cell frequencies and diminished Treg numbers, underscoring the pathogenic role of this imbalance (45). The persistence and recurrence of uveitis have been linked to this disrupted Th17/Treg ratio, as Treg dysfunction impairs the resolution of inflammation, while Th17 cells drive ongoing immune activation.

Patterns of Treg dysfunction exhibit variability across different subtypes of uveitis. In the context of HLA-B27-associated uveitis, Zhuang et al. identified an imbalance between Th17 and Treg cells, characterized by an increase in Th17 cells, a decrease in Treg cells, and a positive correlation with disease activity (3). In VKH disease, metabolomic analyses have demonstrated an enrichment of oleic acid, which influences CD4+ T cell differentiation through the ODC1-STAT5A axis (refer to Section 5.2) (46). Uveitis associated with Behçet’s disease is characterized by Th1/Th17-driven pathology, marked by elevated levels of IFN-γ and IL-17, indicating distinct inflammatory pathways that contribute to Treg exhaustion (47, 48). These subtype-specific differences hold significant implications for targeted therapeutic interventions.

The regulation of the Th17/Treg balance is complex and involves several interrelated mechanisms, including cellular plasticity, cytokine milieu, and microRNA (miRNA)-mediated epigenetic control. Notably, Th17 and Treg cells exhibit lineage plasticity under certain microenvironmental conditions, influenced by cytokines such as TGF-β, IL-6, IL-23, and IL-10. This plasticity allows for dynamic shifts in immune responses during uveitis, where pro-inflammatory signals promote Th17 differentiation, while anti-inflammatory factors favor Treg induction (4). Moreover, miRNAs have emerged as key regulators of this balance. For instance, miR-30b-5p suppresses Notch signaling and Th17 differentiation while promoting Treg expansion, thereby restoring immune homeostasis in EAU (49, 50). Similarly, miR-19b-3p carried by plasma-derived exosomes from Behçet’s uveitis patients disrupts the Th17/Treg balance by downregulating CD46 expression, highlighting the role of extracellular vesicle-mediated miRNA transfer in modulating T cell subsets (48). These findings suggest that targeting miRNA pathways could offer novel therapeutic avenues.

Pharmacological interventions have also demonstrated efficacy in modulating the Th17/Treg axis. Kurarinone, a bioactive compound from traditional Chinese medicine, ameliorates EAU by inhibiting Rac1 and the Id2/Pim1 axis, thereby suppressing Th17 pathogenicity and enhancing Treg function (51). Similarly, prednisone acetate restores Th17/Treg balance through Notch signaling inhibition, reducing ocular inflammation in EAU (52). Other agents such as apremilast regulate this balance via the PI3K/AKT/FoxO1 pathway (53), dipyridamole through the cAMP-STAT3-PIM1 axis (54), and sinomenine by suppressing PI3K/AKT and NF-κB pathways (10). Dietary interventions like caloric restriction and ketogenic diet also favor Treg proliferation and inhibit Th17 expansion, indicating metabolic modulation as a therapeutic strategy (55, 56).

The molecular underpinnings of Th17/Treg imbalance involve transcription factors such as RORγt for Th17 and Foxp3 for Tregs, whose expression is influenced by signaling pathways including STAT3, Notch, and PI3K/AKT. Dysregulation of these pathways leads to enhanced Th17 differentiation and impaired Treg stability. For example, PIM1 kinase enhances Th17 pathogenicity via AKT/FOXO1 signaling, and its inhibition reduces Th17 cells while increasing Tregs (57). Additionally, epigenetic modifications, such as DNA hydroxymethylation changes in genes like TUBB4B, influence Th17/Th1/Treg balance in Behçet’s uveitis (58).

In summary, the disruption of the Th17/Treg balance is a central pathogenic mechanism in uveitis, characterized by increased pro-inflammatory Th17 cells and decreased immunosuppressive Tregs. This imbalance is regulated by complex cellular plasticity, cytokine networks, miRNA-mediated epigenetic control, and intracellular signaling pathways. Therapeutic strategies aiming to restore this balance—through pharmacological agents, miRNA modulation, or metabolic interventions—hold promise for effective management of uveitis by suppressing inflammation and promoting immune tolerance.

In addition to the Th17/Treg imbalance, recent evidence highlights specific signaling pathways that play a crucial role in regulating Treg fitness within the ocular environment. Notably, the adenosine A2Ar signaling axis has been identified as a key pathway involved in both the induction and functional maintenance of Treg cells.

## 3 The role of molecular signaling pathways and metabolic regulation in Tregs exhaustion

### 3.1 Defective A2Ar signaling and impaired maintenance/induction of suppressive Tregs in uveitis

The adenosine A2A receptor (A2Ar) serves as an immunoregulatory pathway that facilitates and stabilizes Treg programs via cAMP/PKA/CREB signaling. Additionally, it enhances the CD39/CD73–adenosine axis, thereby promoting FOXP3 expression and augmenting suppressive function.

Evidence pertinent to uveitis suggests that the A2Ar-dependent induction and/or maintenance of suppressive Treg programs is compromised in autoimmune contexts. In EAU, research utilizing FoxP3-GFP-Cre reporter mice has identified a subset of TIGIT+ Tregs, which are relevant to the disease. The generation of these Tregs necessitates A2Ar activation and is crucial for the effective resolution of ocular inflammation (59). Notably, the induction of TIGIT+ Tregs driven by A2Ar is robust in healthy individuals but is impaired in patients with autoimmune diseases, including uveitis. This observation aligns with the hypothesis of a defective A2Ar-mediated regulatory induction pathway in human autoimmunity (38, 59). Collectively, these data endorse a uveitis-centric model wherein insufficient A2Ar-mediated generation and localization of TIGIT+ Tregs contribute to sustained ocular inflammation. This paradigm elucidates why systemic immune regulation may seem quantitatively intact in certain patients, yet ocular inflammation persists—suggesting that Tregs may be functionally or spatially inadequate at critical ocular sites.

While numerous mechanistic details have been elucidated primarily in non-ocular disease contexts, they offer a credible foundation for understanding the observations in uveitis. In conditions such as sepsis and other inflammatory environments, activation of the A2Ar enhances FOXP3 expression through CREB phosphorylation, thereby promoting the stability of Tregs (60). Concurrently, A2Ar signaling interacts with the expression and function of the ectonucleotidases CD39 and CD73, which convert extracellular ATP to adenosine, potentially maintaining suppression through autocrine and paracrine feedback loops (61, 62). Although direct validation is required to determine whether the cAMP/PKA/CREB–FOXP3 axis and/or CD39/CD73-mediated adenosine production are quantitatively diminished within ocular tissue or aqueous humor during active uveitis, these pathways present viable mechanistic links between impaired A2Ar responsiveness and the reduced suppressive capacity of Tregs in uveitis.

While A2A receptor agonists have demonstrated efficacy in expanding Tregs and suppressing inflammation across various non-ocular models of immune-mediated diseases—such as graft-versus-host disease (63) and *in vitro* systems for peripheral tolerance induction (64)—their application to uveitis requires careful consideration of endpoints pertinent to ocular health. These endpoints include the improvement of EAU clinical and histological scores, direct evidence of increased induction of TIGIT^+^ and/or PD-1^+^ Tregs, normalization of the Th17/Treg balance, and enhanced trafficking or retention of Tregs within ocular tissues or draining lymphoid compartments. Conversely, the ability of A2A receptor blockade to enhance effector immunity, as utilized in cancer immunotherapy, underscores the context-dependent nature of this pathway and emphasizes the necessity for thorough safety evaluations when considering A2A receptor agonists for the treatment of inflammatory eye diseases (65, 66).

In summary, evidence from uveitis and EAU indicates that impaired A2A receptor function may hinder TIGIT+ regulatory T cells, affecting immune regulation in the eye. However, this evidence is limited by model dependence and variability in timing and compartments. Non-ocular studies suggest that disruptions in the cAMP/PKA/CREB pathway or the CD39/CD73–adenosine loop might be involved, but these need confirmation in ocular settings. Future research should focus on profiling the A2A–TIGIT axis in both peripheral and ocular contexts and testing if A2A modulation can enhance Treg function and reduce eye inflammation.

Concurrently with the regulation mediated by A2Ar, the Notch signaling pathway has emerged as a key regulator of T cell fate decisions in uveitis, with significant implications for the Th17/Treg axis.

### 3.2 Notch signaling pathway and miR-30b-5p regulation

The Notch signaling pathway plays a fundamental role in immune regulation, particularly influencing the differentiation of T helper (Th) cells and Tregs, which are crucial in the pathogenesis of autoimmune uveitis. Notch1 and its ligand DLL4 are crucial in promoting Th17 cell development, leading to ocular inflammation. miR-30b-5p acts as an epigenetic regulator by targeting and suppressing Notch1 and DLL4, thereby inhibiting Notch signaling. This suppression restores the Th17/Treg balance, reducing Th17 differentiation and increasing Treg cells, which alleviates inflammation in EAU. This mechanistic insight underscores miR-30b-5p as a promising therapeutic target for restoring immune homeostasis in uveitis through modulation of Notch-mediated Th cell differentiation (49).

Complementing these molecular findings, traditional Chinese medicine formulations, such as Longdan Xiegan Decoction (LXD), have been shown to exert immunomodulatory effects in uveitis by regulating miR-30b-5p expression. Network pharmacology analyses reveal that bioactive compounds within LXD enhance miR-30b-5p levels, thereby inhibiting Notch signaling activation and suppressing the commitment of uveitogenic T-cell lineages. This results in a rebalanced Th1/Th2 and Th17/Treg cell ratio, with decreased proinflammatory cytokines and increased anti-inflammatory cytokines, ultimately reducing ocular inflammation and tissue damage. The therapeutic efficacy of LXD is corroborated by *in vivo* imaging and histopathological assessments in EAU models, which demonstrate significant attenuation of inflammation following treatment. These findings suggest that LXD mediates its beneficial effects at least partly through the miR-30b-5p/Notch1/DLL4 axis, offering a novel integrative approach to immune regulation in uveitis. The capacity of LXD to modulate miRNA expression and downstream signaling pathways highlights the potential of combining traditional herbal medicine with molecular-targeted strategies to achieve immune homeostasis in autoimmune ocular diseases (50).

Moreover, the regulatory role of miR-30b-5p in controlling Notch signaling extends beyond immune cell differentiation to influence vascular and tissue remodeling processes. Studies in pulmonary hypertension models demonstrate that decreased miR-30b-5p levels lead to overexpression of DLL4, impairing angiogenesis through dysregulated Notch signaling. Restoration of miR-30b-5p improves endothelial function by balancing DLL4 and Jagged1 ligands, which may have parallels in the vascular inflammation observed in uveitis. Although these findings stem from non-ocular models, they provide additional evidence for the epigenetic control exerted by miR-30b-5p on Notch pathway components, reinforcing its therapeutic relevance in inflammatory diseases characterized by aberrant Notch activation (67).

In summary, the interplay between Notch signaling and miR-30b-5p is central to the immunopathogenesis of uveitis, where Notch activation favors proinflammatory Th17 differentiation and Treg suppression. miR-30b-5p counteracts this by targeting Notch1 and DLL4, restoring Th17/Treg balance and alleviating inflammation. Traditional Chinese medicine such as Longdan Xiegan Decoction harnesses this mechanism to achieve immune modulation, highlighting a promising avenue for integrated therapeutic strategies. Continued exploration of miR-30b-5p-mediated Notch regulation may yield novel interventions to control autoimmune uveitis and related inflammatory disorders.

## 4 The impact of microenvironmental factors on Tregs exhaustion

### 4.1 Inflammatory microenvironment and Tregs plasticity

The inflammatory microenvironment exerts a profound influence on the stability and plasticity of Tregs, often driving their phenotypic conversion toward pro-inflammatory states that can exacerbate disease progression. Tregs, characterized by the expression of the transcription factor FOXP3, are important for maintaining immune homeostasis and self-tolerance by suppressing excessive immune responses. However, in cytokine-rich inflammatory milieus, Tregs demonstrate considerable plasticity, losing their suppressive function and acquiring effector-like properties that contribute paradoxically to inflammation and tissue damage (68, 69). This plasticity is exemplified by the dynamic interconversion between Tregs and Th17 cells, two CD4+ T cell subsets with opposing immunological roles. Under specific inflammatory conditions, Tregs can transdifferentiate into Th17-like cells, characterized by the production of pro-inflammatory cytokines such as IL-17, thereby promoting disease progression in autoimmune and inflammatory disorders (68, 70).

Mechanistically, the inflammatory cytokine milieu, including IL-1β, IL-6, and TGF-β, orchestrates epigenetic and transcriptional reprogramming of Tregs, destabilizing FOXP3 expression and facilitating their conversion into pathogenic phenotypes (71). For instance, the Hedgehog signaling pathway has been implicated in maintaining Treg identity and suppressive function; its inhibition leads to metabolic rewiring that favors a Th17-like inflammatory profile (70). Furthermore, transcriptional regulators such as the Ikaros family and Notch signaling play critical roles in maintaining the Th17/Treg balance by modulating gene expression programs that govern T cell fate decisions within the inflammatory microenvironment (71, 72).

The reciprocal plasticity between Tregs and Th17 cells is not only a hallmark of autoimmune diseases but also a significant factor in tumor immunity, where Tregs often adopt a more stable and suppressive phenotype to inhibit anti-tumor responses (73). However, in chronic inflammatory conditions, Tregs may lose stability, leading to a reduction in their immunosuppressive capacity and an increase in local inflammation. This phenomenon is evident in diseases such as autoimmune uveitis, where Treg instability and conversion to pro-inflammatory phenotypes contribute to ocular tissue damage (74). Moreover, the gut microbiota and dysbiosis significantly influence Treg plasticity by modulating the local cytokine milieu and metabolic environment, further affecting Treg stability and function (75).

Therapeutic strategies aimed at stabilizing Tregs or modulating their plasticity have shown promise in experimental models. For example, delivery systems such as hyaluronan methylcellulose hydrogels enhance Treg survival and stability in inflammatory settings, improving therapeutic outcomes in autoimmune uveitis (13). Additionally, pharmacological modulation of signaling pathways like Notch and eIF5A can promote the conversion of inflammatory T helper cells toward regulatory phenotypes, restoring immune balance (76). Understanding the molecular determinants of Treg plasticity within the inflammatory microenvironment thus provides critical insights for developing targeted interventions in autoimmune diseases, cancer, and chronic inflammatory disorders.

Inflammatory cytokines present within the ocular microenvironment directly influence the stability of Tregs, while systemic factors, including circadian rhythms, significantly impact Treg metabolism and functionality.

### 4.2 Circadian disruption–driven metabolic insufficiency and Treg functional decline

Circadian rhythms, the intrinsic 24-hour biological cycles, profoundly influence immune system function, including the regulation of Tregs, which are essential in maintaining immune tolerance and preventing autoimmunity such as uveitis. A recent study using a light-induced circadian rhythm disruption model demonstrated that disruption of circadian rhythms exacerbates autoimmune uveitis by impairing Treg stability and immunosuppressive function. Mechanistically, Per1 expression is significantly reduced under circadian disruption, leading to compromised Treg metabolism and diminished immune regulation, thereby aggravating disease progression (77). This finding underscores the essential role of Per1 in maintaining Treg homeostasis and highlights how circadian rhythm integrity supports Treg-mediated immune suppression in autoimmune conditions.

Beyond Per1, other circadian clock components also contribute to Treg function and immune homeostasis. For example, visceral adipose tissue (VAT) Tregs exhibit cell-intrinsic circadian oscillations regulated by core clock genes such as BMAL1. BMAL1 deficiency in Tregs leads to their constitutive activation, metabolic dysfunction, and reduced fitness, resulting in increased tissue inflammation. This suggests that circadian clock genes optimize Treg function and survival by regulating their activation states and metabolic pathways in a tissue-specific manner (78). Such diurnal regulation of Tregs may extend to other tissues, including ocular tissues affected in uveitis, although direct evidence remains to be elucidated.

Circadian disruption also broadly influences immune senescence and inflammatory responses, which can indirectly affect Treg populations. Chronic circadian misalignment accelerates immune aging and chronic inflammation, as shown by increased markers of immune senescence and inflammatory cell infiltration in multiple organs (79). These systemic immune alterations may further destabilize Treg populations and impair their suppressive capacity, contributing to heightened autoimmune responses.

The clinical relevance of circadian regulation of Tregs is further supported by observations that circadian disruption exacerbates inflammatory diseases. In murine models of allergic airway inflammation and autoimmune uveitis, circadian rhythm disruption leads to enhanced proinflammatory Th2 and Th17 responses alongside a reduction in Treg numbers and function, worsening disease severity (80, 81). Additionally, circadian proteins such as REV-ERBα and REV-ERBβ regulate immune cell identity and plasticity, including the maintenance of group 3 innate lymphoid cells (ILC3s) and suppression of proinflammatory cytokine production, which are critical for tissue homeostasis (82). These findings collectively highlight the importance of circadian biology in shaping the immune landscape, particularly by modulating Treg-mediated immune tolerance.

**Figure 3:**
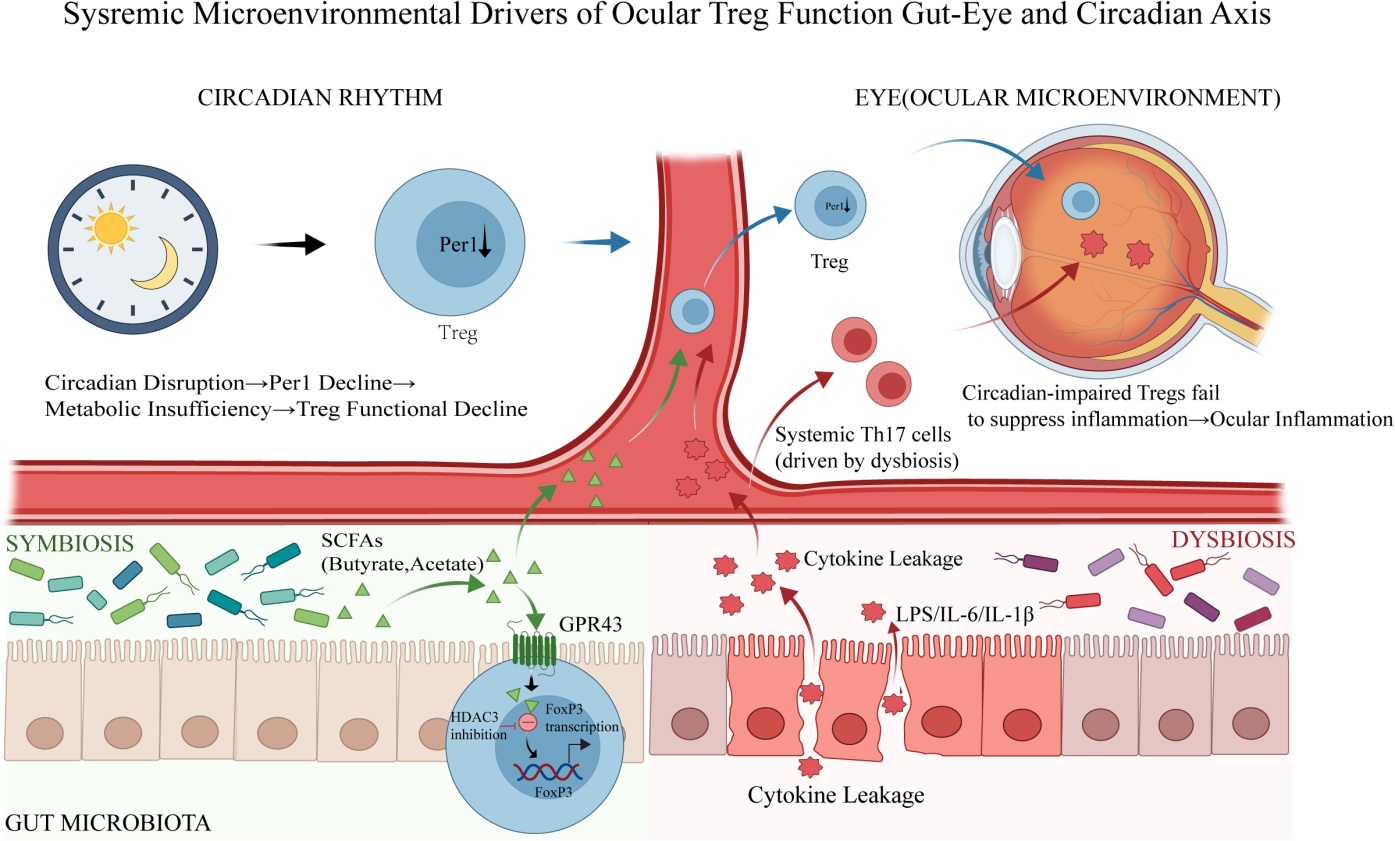
Systemic microenvironmental drivers of ocular Treg function: the gut–eye and circadian axes. Schematic illustrating how systemic cues shape Treg fitness and inflammatory balance in the ocular microenvironment. Circadian axis (top left): Light/dark–entrained circadian signaling maintains Treg function in part through the clock gene Per1; circadian disruption is proposed to reduce Per1 expression, causing metabolic insufficiency and a decline in Treg suppressive capacity. Gut microbiota axis (bottom): During symbiosis, commensal microbiota-derived short-chain fatty acids (SCFAs; e.g., butyrate and acetate) enter the circulation and signal through GPR43 on Tregs, leading to HDAC3 inhibition and enhanced FoxP3 transcription, thereby supporting Treg stability. In contrast, dysbiosis compromises epithelial barrier integrity, promoting translocation of microbial products and inflammatory mediators (LPS, IL−6, IL−1β) into the bloodstream (“cytokine leakage”). Eye (top right): Systemic inflammation associated with dysbiosis facilitates Th17 skewing and trafficking of pathogenic Th17 cells to the eye; concurrently, circadian-impaired Tregs fail to adequately restrain these responses, culminating in ocular inflammation. Green symbols denote SCFAs, whereas red symbols indicate pro-inflammatory mediators in systemic circulation. FoxP3, forkhead box P3; GPR43, G protein−coupled receptor 43; HDAC3, histone deacetylase 3; IL, interleukin; LPS, lipopolysaccharide; Per1, period circadian regulator 1; SCFAs, short−chain fatty acids; Th17, T helper 17 cell; Treg, regulatory T cell.

Because circadian programs intersect with systemic metabolic cues shaped by the gut microbiota, we summarize these connected extrinsic drivers in **Figure 3** and then discuss the gut–eye axis in the next section.

Circadian rhythms are vital for Treg metabolism and function, with the Per1 gene being essential for their metabolic health. Disrupting these rhythms can worsen autoimmune uveitis and other inflammatory diseases. Most evidence comes from experimental studies, with limited human data linking circadian disruption to eye health, complicated by treatment and lifestyle factors. Circadian-based interventions like sleep optimization or melatonin may support Treg health but should be supplementary and clinically validated using uveitis-specific outcomes, considering chronotype and medication timing, before being recommended for uveitis treatment.

### 4.3 Gut microbiota and Tregs development

Emerging evidence increasingly supports the concept of a “gut–eye axis” in the context of non-infectious uveitis, suggesting that intestinal dysbiosis may alter systemic T cell polarization and subsequently affect ocular immune privilege (83, 84). Within uveitis-related contexts, the most consistently observed immunological connection is the Th17/Treg axis. Dysbiosis-associated reductions in Treg-inducing metabolites, such as short-chain fatty acids (SCFAs), and/or increases in pro-inflammatory signals can promote Th17 dominance (85, 86). This phenomenon is a hallmark observed in both human uveitis samples, including aqueous humor and peripheral blood mononuclear cell (PBMC) profiling, as well as in experimental autoimmune uveitis (44, 45). However, direct causal evidence in humans remains limited. Therefore, current findings should be viewed as mechanistic plausibility informed by systemic autoimmune literature, rather than definitive proof of microbiota-driven uveitis in all patients.

Importantly, Mucosal homeostasis critically depends on the coordinated activity of Th17 and Treg cells. Th17 cells produce IL-17 and IL-22, which facilitate epithelial repair and the production of antimicrobial peptides. In contrast, Treg cells, through the secretion of IL-10 and TGF-β and the regulation of antigen-presenting cells via CTLA-4, mitigate excessive inflammatory responses against commensal microorganisms (87). In instances of dysbiosis and barrier disruption, microbial products such as lipopolysaccharide (LPS) and cytokines like IL-6, IL-1β, and IL-23 can infiltrate systemic circulation, a phenomenon referred to as “cytokine leakage” (88). This condition promotes the polarization of pathogenic Th17 cells and compromises the stability of Treg cells. The resulting systemic environment, skewed towards Th17 dominance, is mechanistically associated with the pathogenesis of uveitis. Circulating Th17 cells can migrate to ocular tissues, exacerbating local inflammation, while the diminished or unstable Treg population fails to maintain ocular immune privilege.

The interaction between gut microbiota and Tregs is bidirectional; Tregs modulate antigen-specific immune responses to gut microbes, facilitating a symbiotic relationship that preserves mucosal integrity and systemic immune tolerance (87). Dysbiosis, or imbalance in gut microbial communities, disrupts this delicate equilibrium, leading to diminished Treg populations and impaired function, which exacerbates immune dysregulation in various inflammatory and autoimmune diseases, including uveitis. Experimental models have demonstrated that specific microbial strains, such as Lactobacillus and Faecalibacterium prausnitzii, are potent inducers of Tregs, and their depletion correlates with increased inflammation (89, 90).

Therapeutic interventions targeting the gut microbiota, such as probiotics, prebiotics, postbiotics, and fecal microbiota transplantation (FMT), have shown promise in restoring Treg-mediated immune homeostasis and ameliorating inflammation in both intestinal and extraintestinal contexts (88, 91). In the setting of uveitis, where immune privilege is compromised and Treg exhaustion contributes to pathology, gut microbiota dysbiosis may exacerbate immune imbalance by impairing Treg development and function. This is supported by evidence that microbial metabolites like p-coumaric acid promote peripheral Treg differentiation via modulation of signaling pathways such as PKCθ-AKT-FoxO1/3a, enhancing Treg abundance and function in distant tissues (92). Furthermore, alterations in gut microbiota composition have been linked to the severity of autoimmune conditions through their impact on the Th17/Treg axis, underscoring the critical role of microbial communities in shaping systemic immune responses (83, 84).

These findings highlight the role of gut microbiota in regulating Treg cells and immune tolerance. However, due to limited evidence specific to uveitis, individual differences, and confounding factors like diet and treatments, microbiota-targeted therapies such as probiotics and fecal transplants should be considered experimental for uveitis. Many studies lack functional insights and ocular endpoints. More rigorous, longitudinal research incorporating metabolomics and treatment stratification is needed to assess if microbiota modulation can improve uveitis outcomes beyond systemic immune effects.

## 5 Advances in Tregs-based therapeutic strategies

In order to prevent the overemphasis on conceptually appealing yet clinically underdeveloped interventions, we categorize proposed strategies based on their translational maturity and anticipated clinical application in the management of uveitis. Tier 1 strategies are characterized by robust mechanistic justification and direct efficacy signals in EAU, positioning them as the most viable candidates for disease-modifying interventions aimed at acute control or as steroid-sparing alternatives. Examples include checkpoint agonists that enhance suppressive Treg pathways, such as TIGIT-centered mechanisms in EAU, targeted small molecules with proven efficacy in uveitis models, and localized delivery systems designed to enrich or stabilize Tregs within ocular tissues. Conversely, Tier 2 strategies possess supportive immunometabolic or immunoregulatory rationale but lack substantial uveitis-specific clinical evidence. These should primarily be considered as adjunctive, maintenance, or preventive measures rather than independent disease-modifying therapies. Examples include interventions targeting circadian rhythm alignment, dietary patterns, and microbiome modulation.

**Table 1:** Subtype−specific characteristics of Treg dysfunction in non−infectious uveitis.

Uveitis subtype	Primary treg abnormalities	Key immunological features	Metabolic signature	Therapeutic implications
Behçet’s disease	Quantitative reduction; impaired suppressive function	Th1/Th17-driven pathology; elevated IFN−γ and IL−17; distinct T cell clonality	Enhanced glycolytic flux	Anti−TNF agents; IL−17/IL−23 pathway inhibitors
VKH disease	Lineage instability; Th17−skewed plasticity	Melanocyte−specific autoimmunity; distinct T cell phenotypes vs. BD	Oleic acid enrichment; OA−ODC1−STAT5A pathway involvement	ODC1 inhibition; metabolic reprogramming; corticosteroids
HLA−B27−associated uveitis	Th17−skewed imbalance	IL−23/IL−17 axis activation; genetic predisposition	Enhanced glycolytic flux	IL−17/IL−23 pathway inhibitors

To improve readability and facilitate clinical translation, we summarized currently used and emerging therapeutic options in non-infectious uveitis with their immunological mechanisms and reported outcomes (**Table 2**), and representative clinical trials/clinical-stage studies relevant to uveitis treatment and Treg-related immunomodulation (**Table 3**).

**Table 2:** Treg-/Th17–Treg axis–oriented therapeutic strategies relevant to non-infectious uveitis.

Strategy	Representative intervention	Core Treg/Th17–Treg mechanism	Evidence (uveitis/EAU/other)	Key finding	Ref.
Checkpoint agonism	TIGIT stimulation/agonist	Enhances suppressive TIGIT+ Tregs; limits Th17 infiltration	EAU	Suppressed EAU and reduced Th17 infiltration while improving Treg function	(91)
Immunometabolic reprogramming	Itaconate	Restores Teff/Treg (incl. Th17/Treg) balance via metabolic/epigenetic rewiring	EAU	Ameliorated EAU; corrected Teff/Treg imbalance (DNAJA1/CDC45 axis)	(93)
Dietary intervention	Caloric restriction	Metabolic remodeling (↓PI3K/AKT/c-Myc, ↓glycolysis) → ↑Treg proliferation	EAU	Protected against EAU by improving Teff/Treg balance (scRNA-seq supported)	(51)
Dietary intervention	Ketogenic diet	Immune transcriptional/metabolic shift favoring regulatory balance	EAU	Reduced EAU severity with broad immune transcriptional changes	(52)
miRNA therapy (Notch)	miR-30b-5p lentiviral delivery	Inhibits Notch1/DLL4 → restores Th17/Treg balance	EAU	Therapeutic effect in EAU through Notch inhibition and Th17/Treg rebalance	(44)
Herbal (miRNA–Notch axis)	Longdan Xiegan Decoction (LXD)	Inhibits Notch; upregulates miR-30b-5p → suppresses Th17, supports Treg	EAU	Alleviated EAU and restored Th17/Treg homeostasis	(45, 68)
Small-molecule immunomodulation	Kurarinone	Suppresses Th17 pathogenicity; improves Th17/Treg balance (Rac1; Id2/Pim1)	EAU	Ameliorated autoimmune uveitis by rebalancing Th17/Treg	(47)
Trafficking/immune rebalancing	CXCR3 antagonist AMG487	Restores effector/Treg balance; reduces inflammatory programs	EAU	scRNA-based analyses supported therapeutic effects in EAU	(112)
Exosome-based immunoregulation	IL-27-containing exosomes	Promotes Treg expansion; suppresses Th1/Th17 responses	EAU	Suppressed/ameliorated uveitis via regulatory skewing	(111)
Treg cell therapy enabling tech	Intravitreal Tregs + HA-MC hydrogel	Improves survival/retention/stability of transferred Tregs in eye	EAU	Enhanced efficacy of intravitreal Treg therapy in EAU	(11)
Human endotype/target concept	Exosomal miR-19b-3p (BU)	Drives Th17 dominance and Treg reduction via CD46; inhibition restores balance	Human Behçet’s uveitis	Patient exosomal miR-19b-3p linked to Th17/Treg imbalance; inhibition reverses skewing	(46)

**Table 3:** Representative studies/clinical-stage evidence relevant to uveitis therapy and Treg-directed immunomodulation.

Study focus	Disease/model	Intervention/exposure	Study type/design	Primary readouts	Main outcome	Ref.
Checkpoint agonism to suppress uveitis	EAU	TIGIT stimulation	Preclinical intervention	Clinical score; Th17 infiltration; Treg function/transfer	Reduced EAU; inhibited Th17 infiltration; enhanced Treg-mediated protection	(91)
Immunometabolic therapy	EAU	Itaconate	Preclinical intervention	EAU severity; Teff/Treg balance; mechanism axis	Ameliorated EAU by restoring Teff/Treg imbalance (DNAJA1/CDC45)	(93)
Local Treg delivery platform	EAU	Intravitreal Tregs + HA-MC hydrogel	Preclinical translational biomaterials study	Treg persistence/stability; efficacy	Improved efficacy of intravitreal Treg therapy	(11)
Dietary caloric restriction	EAU	CR	Preclinical + scRNA-seq	Teff/Treg ratio; metabolic pathways	Increased Treg proliferation; protected against EAU	(51)
Ketogenic diet	EAU	KD	Preclinical	Immune transcriptional landscape; severity	KD ameliorated EAU with immune remodeling	(52)
miRNA therapy targeting Notch	EAU	miR-30b-5p lentivirus	Preclinical gene/miRNA therapy	Notch activation; Th17/Treg balance; severity	Improved EAU by inhibiting Notch and restoring Th17/Treg balance	(44)
Human immune profiling (Th17/Treg axis)	Intermediate uveitis	—	Clinical observational immune profiling	PBMC/aqueous Th17 & Treg; cytokines	Th17/Treg dysregulation correlated with local/systemic immune response	(42)
Human mechanistic target (exosomal miRNA)	Behçet’s uveitis	Exosomal miR-19b-3p axis	Patient-sample translational study	Th17/Treg balance; CD46	Exosomal miR-19b-3p associated with Th17/Treg imbalance; inhibition restores balance (mechanistic)	(46)
Related clinical paradigm (Treg expansion)	SLE	Low-dose IL-2	Clinical evidence summarized (review)	Treg expansion; disease activity (as summarized)	Supports feasibility of selective Treg expansion in autoimmunity	(10)

### 5.1 Immune checkpoint agonists and small molecule drugs

The use of immune checkpoint agonists and small molecule drugs represents a promising frontier in the treatment of uveitis by modulating the balance and function of T cell subsets, particularly Tregs and Th17 cells, which are critical in ocular immune homeostasis and inflammation. Among immune checkpoint agonists, TIGIT (T cell immunoglobulin and ITIM domain) has emerged as a novel target with significant therapeutic potential. TIGIT is an inhibitory receptor expressed on T cells, including Tregs and Th17 cells, acting to suppress T cell activation and promote immune tolerance. EAU, a murine model of human uveitis, has been used to evaluate the effects of TIGIT stimulation. Administration of a TIGIT agonist at disease onset markedly reduced the severity of uveitis and inhibited the infiltration of pathogenic Th17 cells into ocular tissues. This effect was accompanied by enhanced suppressive function of Tregs, which were capable of transferring resistance to uveitis upon adoptive transfer, indicating that TIGIT stimulation not only suppresses effector T cell-mediated inflammation but also promotes regulatory immunity (93). These findings underscore TIGIT agonists as a promising immunotherapeutic strategy that could restore immune tolerance in uveitis by rebalancing the Th17/Treg axis. TIGIT agonism is proposed as a strategy to enhance the function or stability of TIGIT+ Tregs and to prevent early dysfunction, rather than trying to activate terminally exhausted, non-functional cells.

In addition to immune checkpoint agonists, small molecule drugs that regulate metabolic and signaling pathways have demonstrated potential in restoring immune homeostasis in inflammatory diseases, including uveitis. Itaconate, a metabolite derived from the tricarboxylic acid cycle, has been recognized for its anti-inflammatory properties through modulation of cellular metabolism and redox balance. By influencing metabolic reprogramming, itaconate can suppress proinflammatory Th17 responses while enhancing Treg differentiation, thereby restoring the Th17/Treg balance critical for controlling autoimmune inflammation (94). A seminal study conducted in 2024 revealed that itaconate treatment markedly reduced the severity of EAU (95). This was achieved through the reprogramming of inflammatory immunometabolism and the restoration of the Th17/Treg balance within ocular tissues. These findings, specific to uveitis, establish itaconate as a mechanistically substantiated candidate for immunometabolic intervention.

Traditional herbal formulations have been investigated in the context of experimental autoimmune uveitis. Notably, Longdan Xiegan Decoction has demonstrated efficacy in ameliorating EAU in rat models. This therapeutic effect is achieved through the inhibition of Notch signaling and Th17 cell differentiation, alongside the restoration of the Th17/Treg cell balance (72). These findings are in alignment with later mechanistic studies that highlight the involvement of the miR-30b-5p/Notch axis (50).

In summary, TIGIT agonists show promise in suppressing Th17-driven ocular inflammation and supporting Treg function, but their safety for uveitis remains uncertain due to preclinical evidence and concerns about infection risk and long-term immune suppression. Similarly, small molecules like itaconate derivatives and herbal formulations exhibit potential in rebalancing Th17/Treg responses, but issues with bioavailability, target engagement, and drug interactions hinder clinical use. Future research should focus on identifying key cellular targets, establishing biomarkers for patient stratification, and conducting trials to optimize dosing and assess the efficacy and safety of these treatments, both alone and with standard immunosuppression.

### 5.2 Dietary intervention and metabolic regulation

Dietary interventions, particularly ketogenic diets (KDs), have emerged as influential modulators of immune cell function, including the differentiation and activity of Tregs, which play a pivotal role in maintaining immune tolerance and controlling inflammation in autoimmune diseases such as uveitis. The ketogenic diet, characterized by high fat and low carbohydrate intake, induces profound metabolic shifts that affect immune cell transcriptional programs and function. Recent studies have elucidated that KD promotes Treg differentiation by modulating fatty acid metabolism pathways. Specifically, the enzyme stearoyl-CoA desaturase-1 (SCD1), which catalyzes fatty acid desaturation and is sensitive to dietary influences, acts as a negative regulator of Treg differentiation. Inhibition or genetic ablation of SCD1 enhances the hydrolysis of triglycerides and phosphatidylcholine via adipose triglyceride lipase (ATGL), leading to increased release of docosahexaenoic acid (DHA). DHA activates peroxisome proliferator-activated receptor gamma (PPARγ), a nuclear receptor that promotes Treg differentiation and suppresses autoimmunity, as demonstrated in models of multiple sclerosis (96). This mechanistic insight underscores how ketogenic diets can reprogram immune metabolism to favor Treg-mediated immunosuppression, potentially alleviating ocular inflammation in uveitis.

Caloric restriction (CR), a dietary intervention that reduces overall caloric intake without malnutrition, has been shown to increase Treg populations and suppress pro-inflammatory Th1 and Th17 cells in EAU. Single-cell RNA sequencing analyses revealed that CR enhances Treg proliferation by altering immune cell metabolism, notably downregulating glycolysis and inflammatory gene expression, and inhibiting the PI3K/AKT/c-Myc signaling pathway. This metabolic remodeling results in a balanced Teff/Treg ratio and mitigates ocular inflammation (55). These findings highlight the potential of metabolic interventions to restore immune homeostasis through Treg modulation.

The role of gut microbiota-derived metabolites, particularly short-chain fatty acids (SCFAs) such as butyrate, propionate, and acetate, is also critical in regulating Treg function and systemic immune responses. Dietary components influence the composition and metabolic activity of the gut microbiota, which in turn modulates Treg differentiation and stability. For instance, Lactiplantibacillus plantarum BD7807 increases intestinal SCFA availability, which can activate GPR43 signaling and inhibit HDAC3, thereby promoting tight junction integrity (e.g., ZO-1/occludin/claudin maintenance) and creating an epigenetic environment permissive for FOXP3 transcription and Treg expansion (85). Additionally, dietary supplementation with sodium propionate has been shown to improve lipid metabolism and reduce pro-inflammatory cytokines in people living with HIV, concomitant with changes in Treg populations and gut microbiome composition, suggesting a systemic immunometabolic benefit (97). These data collectively emphasize that diet-induced metabolic regulation provides a novel perspective for immunotherapy in uveitis by targeting Treg metabolism and function.

In addition to the overarching influence of dietary patterns on Treg differentiation, specific dietary fatty acids have the capacity to directly influence CD4+ T cell fate decisions via well-defined molecular pathways. A notable instance is oleic acid (OA), a monounsaturated omega-9 fatty acid, which is found in elevated levels in patients with VKH disease. Research by Liao et al. has elucidated that OA directly interacts with ornithine decarboxylase 1 (ODC1) at the lysine-78 residue, thereby augmenting ODC1 enzymatic activity and facilitating putrescine synthesis (46). This biochemical interaction results in the inhibition of STAT5A phosphorylation at Tyr694 and a concomitant reduction in IL-10 transcription, ultimately fostering a pro-inflammatory CD4+ T cell phenotype characterized by enhanced Th1/Th17 responses and diminished Treg differentiation. Functional assays have corroborated that OA exacerbates EAU, whereas inhibition of ODC1 rectifies the Th1/Th17-Treg imbalance observed in VKH. This pathway exemplifies the direct impact of a specific dietary fatty acid on ocular autoimmunity at the molecular level, effectively linking dietary regulation, immunometabolic signaling, and uveitis endotyping. Furthermore, it underscores ODC1 as a promising therapeutic target for subtype-specific metabolic interventions.

Dietary interventions like ketogenic diets and caloric restriction can influence immune function by altering Treg metabolism and differentiation through fatty acid desaturation, nuclear receptor activation, and metabolic pathways. Additionally, changes in gut microbiota and their metabolites support Treg-mediated immune tolerance. While these dietary changes could complement traditional therapies, they should not replace immunosuppressive or biologic treatments. Clinical studies are required to evaluate the feasibility, effectiveness, and safety of dietary approaches for uveitis patients, given the limited uveitis-specific evidence. These trials should also track adherence, metabolic side effects, and assess the steroid-sparing benefits alongside standard treatments.

### 5.3 miRNA intervention strategies

MicroRNAs (miRNAs) have emerged as critical regulators of immune homeostasis, particularly in modulating the balance between Tregs and T helper 17 (Th17) cells, which is important in the pathogenesis of autoimmune and inflammatory diseases such as uveitis. Among these, miR-30b-5p and miR-19b-3p have been identified as key miRNAs influencing the Treg/Th17 equilibrium, presenting promising therapeutic targets for restoring immune balance in uveitis. For instance, miR-30b-5p expression is notably decreased in EAU, contributing to the activation of the Notch signaling pathway, which promotes proinflammatory Th17 differentiation and suppresses Treg expansion. Treatment with Longdan Xiegan Decoction (LXD) has been shown to upregulate miR-30b-5p, thereby inhibiting Notch1 and DLL4 expression, downregulating proinflammatory cytokines such as IL-17A and RORγt, and restoring the Th17/Treg balance, culminating in amelioration of uveitis symptoms (50). Similarly, miR-19b-3p has been implicated in Behçet’s uveitis (BU), where plasma-derived exosomes from patients with active disease exhibit elevated levels of miR-19b-3p. This miRNA downregulates CD46 expression in CD4+ T cells, skewing the balance towards Th17 dominance and reducing Treg populations, thereby exacerbating inflammation. Inhibition of miR-19b-3p reverses these effects, restoring Treg/Th17 homeostasis and highlighting its potential as a therapeutic target (48). These findings underscore the pathogenic role of specific miRNAs in disrupting immune equilibrium and suggest that targeted modulation of miR-30b-5p and miR-19b-3p can rebalance Treg and Th17 populations, offering a novel avenue for uveitis treatment.

Beyond targeting individual miRNAs, precision delivery systems employing miRNA vectors or mimics have been developed to achieve controlled modulation of immune responses. For example, engineered dendritic cells expressing miRNA mimics, such as miR-5119, have demonstrated efficacy in reducing immune checkpoint ligand expression (e.g., PD-L1), preventing T cell exhaustion, and enhancing antitumor immunity in breast cancer models, indicating the feasibility of miRNA-based immunomodulation (98). Although this study focuses on cancer, the principle of using miRNA-loaded vectors to modulate immune checkpoints and T cell function is translatable to autoimmune conditions like uveitis, where immune exhaustion and dysregulated T cell subsets are involved. Moreover, extracellular vesicles (exosomes) derived from immune cells can serve as natural carriers for miRNAs, facilitating intercellular communication and immune regulation. Immune cell-derived exosomes have shown promise in restoring functional cytokine-producing T cells and inducing Treg expansion, thereby reestablishing immune homeostasis in severe infections and inflammatory diseases (99). Such vesicle-based delivery systems could be harnessed to transport miR-30b-5p or miR-19b-3p modulators directly to target T cell populations within the ocular microenvironment, minimizing off-target effects and enhancing therapeutic precision.

The therapeutic potential of miRNA intervention is further supported by accumulating evidence that miRNAs regulate multiple layers of T cell biology, including differentiation, proliferation, and exhaustion. For instance, miR-155 and miR-146a modulate Treg stability and function, while miR-15/16 clusters restrict effector Treg differentiation, highlighting the complexity and specificity of miRNA-mediated immune regulation (100, 101). Leveraging this knowledge, miRNA-based therapies can be designed to fine-tune Treg and Th17 cell functions, thus restoring immune tolerance and preventing pathological inflammation in uveitis. Importantly, the use of miRNA mimics or inhibitors delivered via optimized vectors or exosomes allows for temporal and spatial control over miRNA expression, enabling the restoration of immune equilibrium without broadly suppressing immune function.

In summary, miRNA-based interventions targeting miR-30b-5p or miR-19b-3p offer a promising approach to modulate the Th17/Treg axis in uveitis, but evidence is mostly preclinical, and human ocular tissue validation is limited. Challenges include ocular delivery, biodistribution, dose control, off-target effects, immune activation, and exosome product standardization. Future research should focus on validating targets in patient samples, assessing pharmacokinetics and safety with relevant delivery methods, and conducting trials to evaluate uveitis outcomes and steroid-sparing effects alongside standard treatments.

## 6 Existing challenges and future research directions

### 6.1 Tregs therapy: preparation and stability Issues

The clinical application of Tregs as a cellular therapy for autoimmune diseases and transplantation has gained significant attention due to their intrinsic immunosuppressive properties. However, the preparation and maintenance of stable, functional Tregs present substantial technical challenges that limit therapeutic efficacy. One primary obstacle lies in the ex vivo expansion and purification of Tregs. Protocols for isolating Tregs often rely on surface markers such as CD4, CD25, and low CD127 expression, but these markers do not exclusively identify stable Tregs, resulting in heterogeneous cell populations with variable suppressive function (102, 103). Moreover, the *in vitro* expansion process must balance achieving sufficient cell numbers with preserving Treg lineage stability, as repeated stimulation can lead to phenotypic drift or exhaustion in conventional T cells. Interestingly, thymus-derived Tregs (tTregs) have demonstrated superior expansion potential and stability compared to peripheral blood-derived Tregs, with protocols incorporating rapamycin to inhibit contaminating effector T cells and maintain FOXP3 expression (104). Despite these advances, ensuring the purity and suppressive function of expanded Tregs remains complex, requiring rigorous quality control including assessments of FOXP3 expression, Treg-specific demethylated region (TSDR) status, and suppressive assays (105, 106).

Another critical issue is the plasticity of Tregs under inflammatory conditions, which can induce their conversion into pro-inflammatory phenotypes, thereby limiting therapeutic benefit. This cellular plasticity is influenced by the microenvironment and metabolic cues; for example, exposure to pro-inflammatory cytokines or hypoxic conditions can destabilize FOXP3 expression and promote effector T cell-like functions (107). Strategies to enhance Treg stability include genetic engineering approaches such as co-expression of FOXP3 with chimeric antigen receptors (CARs), which have been shown to improve lineage fidelity and suppressive capacity even in hostile inflammatory milieus (108). Additionally, metabolic modulation, such as the inclusion of lactic acid during expansion, has been reported to improve Treg viability and reduce exhaustion marker expression, further supporting functional stability (109). Epigenetic regulation also plays a pivotal role; agents like vitamin C derivatives can promote FOXP3 gene demethylation, enhancing Treg suppressive function and stability (110). Despite these promising approaches, the risk of Treg conversion to inflammatory phenotypes remains a significant barrier, necessitating ongoing efforts to optimize culture conditions and molecular modifications.

The preparation of Tregs for therapeutic use faces challenges like efficient isolation, expansion, and maintaining function and stability. While GMP-compatible protocols using thymic sources, rapamycin, metabolic conditioning, and genetic engineering have enhanced Treg quality and quantity, their plasticity under stress limits clinical efficacy. Future strategies should focus on molecular fingerprinting, epigenetic stabilization, and innovative engineering to create stable Treg products for sustained immunomodulation in diseases like uveitis and autoimmune conditions.

### 6.2 Clinical translation: safety and efficacy evaluation

The clinical application of regulatory T cell (Treg)-based therapies for uveitis is still in its nascent stages, characterized by a significant paucity of large-scale clinical trials necessary to thoroughly assess their safety and long-term efficacy. The current body of evidence is largely derived from preclinical models and early-phase studies, which, although promising, do not adequately address the complexities of human immune responses or the potential adverse effects associated with immunomodulation. For example, therapies that target immune checkpoints or cytokine pathways aim to reestablish immune tolerance by enhancing Treg function or numbers; however, these strategies are accompanied by inherent risks. A primary concern is the potential for excessive immunosuppression, which may increase patients’ susceptibility to opportunistic infections or malignancies. Immune checkpoint inhibitors, which have transformed cancer immunotherapy, illustrate this double-edged nature by occasionally inducing immune-related adverse events, including ocular inflammation, while also raising concerns about impaired tumor surveillance when used in other contexts (111). Moreover, the eye’s delicate immune privilege presents challenges for therapeutic interventions, as excessive immune suppression could disrupt local immune homeostasis, thereby heightening the risk of infections or neoplastic transformations.

Recent advances highlight the potential of novel immunotherapies that modulate Treg activity more precisely. For example, IL-27-containing exosomes secreted by innate B-1a regulatory cells have demonstrated efficacy in suppressing uveitis in murine models by promoting Treg expansion and antagonizing pathogenic Th1/Th17 responses without observable adverse effects (112). This approach offers a promising therapeutic avenue by circumventing the dosing challenges of soluble cytokines and potentially minimizing systemic immunosuppression. Similarly, the CXCR3 antagonist AMG487 has shown therapeutic benefit in experimental autoimmune uveitis by restoring the balance between effector T cells and Tregs and reducing inflammatory gene expression in immune cells (113). These findings underscore the importance of targeting specific molecular pathways to achieve immune regulation while limiting off-target effects.

Despite promising preclinical results, translating these therapies into clinical practice requires thorough safety evaluations. There’s a risk that reversing immune exhaustion could unintentionally trigger autoimmunity or cancer, necessitating careful dose optimization and patient selection. The long-term effects of altering Treg populations are uncertain, particularly regarding lasting immune tolerance versus possible immune escape. No large-scale randomized controlled trials have yet confirmed the safety or long-term effectiveness of Treg-based treatments for uveitis, indicating a significant gap in clinical research.

Treg-targeted therapies show promise for treating uveitis by restoring immune balance, but their use is limited by insufficient safety and efficacy data. Future research should focus on rigorous clinical trials and studies to balance therapeutic benefits with risks like infections and malignancies. Overcoming these challenges is crucial to fully utilizing Treg modulation in uveitis and other ocular autoimmune diseases.

## 7 Conclusion

The quantitative reduction and functional exhaustion of Tregs represent a central node in the immunopathogenesis of non-infectious uveitis, driving disease chronicity and recurrence. This review delineates the multidimensional mechanisms compromising Treg fitness, highlighting the loss of FoxP3 stability, aberrant expression of immune checkpoints (e.g., TIGIT and PD-1), and the skewing of the Th17/Treg axis as critical intrinsic drivers. Furthermore, extrinsic microenvironmental factors—including defective adenosine A2A receptor (A2Ar) signaling, circadian rhythm disruption mediated by the clock gene Per1, and alterations in gut microbiota-derived metabolites—exacerbate Treg dysfunction through immunometabolic reprogramming. The identification of the pathogenic oleic acid−ODC1−STAT5A pathway in VKH disease illustrates the direct influence of specific dietary fatty acids on CD4+ T cell differentiation at the molecular level. This finding underscores the evolving paradigm of subtype-specific metabolic interventions in the treatment of uveitis. Collectively, these findings establish an integrated framework linking intracellular molecular networks to systemic microenvironmental cues, elucidating the breakdown of ocular immune tolerance.

Despite this progress, the clinical translation of Treg-centric therapies faces significant hurdles. Future research must prioritize addressing the lineage stability of expanded Tregs to prevent phenotypic conversion within inflammatory milieus. Additionally, rigorous clinical trials are essential to evaluate the safety and long-term efficacy of metabolic and immunomodulatory interventions. By deciphering the heterogeneity of Treg exhaustion and identifying precise biomarkers for patient stratification, these emerging strategies hold the potential to achieve durable inflammation control and vision preservation in uveitis.
